# Risk of SARS‐CoV‐2 Infection and Hospitalisation in Immunocompromised Children: A Population‐Based Cohort Study in Italy and Norway

**DOI:** 10.1111/apa.70509

**Published:** 2026-03-23

**Authors:** Costanza Di Chiara, Riccardo Boracchini, Nhung T. H. Trinh, Arianna Giugni, Giulia Sturniolo, Elisa Visonà, Ali Judd, Marthe Le Prevost, Claire Thorne, Pia Hardelid, Carlo Giaquinto, Angela Lupattelli, Daniele Donà, Anna Cantarutti

**Affiliations:** ^1^ Department for Women's and Children's Health University of Padua Padua Italy; ^2^ Penta – Child Health Research Padua Italy; ^3^ Child Health Evaluative Sciences The Hospital for Sick Children Toronto Ontario Canada; ^4^ Department of Statistics and Quantitative Methods, Division of Biostatistics, Epidemiology and Public Health, Laboratory of Healthcare Research and Pharmacoepidemiology University of Milano‐Bicocca Milan Italy; ^5^ PharmacoEpidemiology and Drug Safety Research Group, Department of Pharmacy, and PharmaTox Strategic Research Initiative University of Oslo Oslo Norway; ^6^ Medical Research Council Clinical Trials Unit at University College London London UK; ^7^ University College London Great Ormond Street Institute of Child Health London UK; ^8^ Centre for Paediatric Epidemiology and Biostatistics, University College London Great Ormond Street Institute of Child Health London UK

**Keywords:** COVID‐19 hospitalisation, COVID‐19 severity, immunocompromised children, population‐based, SARS‐CoV‐2 infection

## Abstract

**Aim:**

Immunocompromised children (IC) were presumed to be at higher risk of SARS‐COV‐2 infection and severe COVID‐19, but population‐based evidence is limited. We assessed infection risk, COVID‐19 hospitalisation, and severe outcomes in IC compared with children with and without other high‐risk conditions.

**Methods:**

We conducted a retrospective population‐based cohort study using electronic health registries from Italy and Norway, including children aged 1–14 years from February 2020–February 2022. Children were classified as IC, non‐immunocompromised with high‐risk conditions (non‐IC), or reference children (RC). Adjusted models estimated infection hospitalisation risks.

**Results:**

We included 29 520 children (IC = 201, non‐IC = 5 144, RC = 24 175) in Italy and 851 517 (IC = 4 862, non‐IC = 157 358, RC = 689 297) in Norway. Infection rates were similar across groups in both countries. Hospitalisations were rare in both datasets; however, in the Norwegian cohort, the aHR for hospitalisation was 5.29 (95% CI: 2.49–11.25) for IC vs. RC and 2.30 (95% CI: 1.10–4.82) for IC vs. non‐IC. Zero and five severe cases occurred in Italy and Norway, respectively.

**Conclusions:**

IC did not have an increased risk of infection but experienced higher hospitalisation rates despite a low incidence of severe disease. This pattern may reflect precautionary clinical management rather than increased disease severity.

**Summary**

Evidence on the risk of SARS‐COV‐2 infection and COVID‐19 outcomes in immunocompromised children is limited, as most previous studies relied on hospitalised cohorts and may be affected by admission bias.

In two population‐based cohorts from Italy and Norway, immunocompromised children had similar infection risk but higher hospitalisation risk, while severe outcomes were rare.

These findings suggest hospitalisation may reflect precautionary clinical management rather than greater disease severity in high‐risk children.

**Abbreviations**
95% CI: 95% confidence interval
aHR: adjusted hazard ratio
ARR: adjusted risk ratio
IC: immuno compromised
ICU: intensive care unit
IQR: interquartile ranges
KUHR: Norway Control and Payment of Health Reimbursement
MSIS: Norwegian Surveillance System for Communicable Disease
Non‐IC: non‐immunocompromised
NPR: Norwegian Patient Registry
RC: reference children
RT‐PCR: reverse transcriptase polymerase chain reaction
SD: standard deviations
SSB: Statistics Norway
SYSVAK: Norwegian Immunisation Registry

## 1 Background

Early in the COVID‐19 pandemic, immunocompromised (IC) children were presumed to have increased risk for severe SARS‐CoV‐2 infection [1], largely based on theoretical concerns rather than paediatric‐specific evidence. While certain underlying chronic conditions—such as cardiovascular, neurologic, and metabolic disorders—have been established as risk factors for severe COVID‐19 in children [2], the same has not been clearly demonstrated for immunocompromised status. Although immunocompromised adults are at increased risk of severe COVID‐19 outcomes [3], current evidence does not support similar associations in paediatric populations [4, 5, 6]. Emerging data suggest that hospitalised IC children are not at increased risk of severe COVID‐19 compared to immunocompetent peers [6, 7]. However, these findings are mainly derived from hospitalised cohorts, where admission practices may reflect clinical caution rather than actual disease severity. This admission bias limits generalizability and may overestimate the true burden in fragile children with immunocompromising conditions.

Evidence on the risk of SARS‐CoV‐2 infection in IC children remains scarce, often limited by small sample sizes [8], and population‐based studies evaluating both infection and hospitalisation risk, and whether hospitalisation correlates with clinical severity, are lacking. A better understanding of these risks is essential to inform evidence‐based clinical management and to guide targeted public health strategies for the care of immunocompromised paediatric populations.

In this population‐based study conducted in Italy and Norway, we assessed the risk of SARS‐CoV‐2 infection and COVID‐19‐related hospitalisation in IC children compared with immunocompetent children, including those with and without other high‐risk underlying conditions. We also examined disease severity among hospitalised cases to provide a clearer picture of the clinical burden in this vulnerable population.

## 2 Material and Methods

### 2.1 Study Design and Data Sources

We conducted a retrospective, population‐based cohort study using two independent paediatric data sources: The Pedianet database in Italy and the linked national health registries in Norway. To account for differences in data structure, coding systems, and healthcare organisations, each dataset was analysed separately. Both sources offer individual‐level, longitudinal data with near‐complete population (Italy) and fully nationwide (Norway) coverage, enabling identification of SARS‐CoV‐2 infections, COVID‐19‐related hospitalisations, and relevant clinical and demographic information.

In Italy, data were drawn from the Pedianet network, a primary care paediatric database compiling routine clinical information from over 200 community‐based paediatricians via the standardised JuniorBit electronic medical record system [9]. Pedianet includes detailed patient‐level data, including demographics, clinical history, symptoms, diagnoses, prescriptions, referrals, and immunisation records, providing a comprehensive view of paediatric health over time. The network covers around 4% of the annual Italian paediatric population and is considered nationally representative [9]. For this study, we focused on data from the Veneto region, where Pedianet is regularly fed to the regional hospital discharge database, SARS‐CoV‐2 nasopharyngeal swab testing registry, and immunisation registry, allowing complete capture of SARS‐CoV‐2 test results (reverse transcriptase polymerase chain reaction [RT‐PCR] and rapid antigen test), COVID‐19‐related hospital admissions, and COVID‐19 vaccination status [10]. This ensured the complete capture of COVID‐19 testing, hospitalisation, and vaccination data.

In Norway, we linked multiple national health databases and administrative databases using unique personal identifiers assigned to all residents. These include the Norwegian Patient Registry (NPR), the Norwegian Control and Payment of Health Reimbursement (KUHR) for information on hospital and primary care encounters, the Norwegian Surveillance System for Communicable Disease (MSIS) for SARS‐CoV‐2 test results, the Norwegian Immunisation Registry (SYSVAK) database for vaccination records, and Statistics Norway (SSB) for population and sociodemographic characteristics [11]. These data sources encompass the entire Norwegian population and provide detailed information on demographics, medical history, diagnoses, procedures, primary care encounters, hospital admissions, test results, and immunisations.

### 2.2 Study Population and Exposure Definition

We included all children aged 1–14 years with continuous residency and valid healthcare data in the respective databases as of 1 February 2020. Age was calculated as of 1 February 2020, corresponding to the study start date. This time frame was selected to capture the period during which community‐level testing for SARS‐CoV‐2 was widely available and mandatory in both countries [12, 13]. Children under one year of age were excluded to ensure accurate ascertainment of chronic underlying conditions, which often require at least 12 months in administrative and primary care data sources [14]. In Pedianet, children were required to be under active follow‐up, defined as having age‐appropriate, documented well‐child visits with their assigned community‐based paediatrician, indicating ongoing clinical care [10]. In Norway, individuals without a valid personal identification number, required for registry linkage, were excluded. This ensured that all individuals included had sufficiently complete data for reliable classification of exposure and outcome status.

Follow‐up began on 1 February 2020, and continued until the outcome of interest (SARS‐CoV‐2 infection and COVID‐19‐related hospitalisation), death, emigration, or study end (28 February 2022), whichever occurred first.

Children were categorised into three mutually exclusive groups based on the presence of chronic or immunocompromising underlying conditions, using diagnostic codes recorded in each data source (ICD‐9‐CM codes in Italy and ICD‐10 and ICPC‐2 codes in Norway), detailed in Tables S1 and S2. Diagnostic codes and free‐text diagnoses were screened and grouped into immunocompromising or non‐immunocompromising conditions by trained paediatricians (CD, GS, and EV), with dual review for ambiguous cases. Specifically, children were classified as: (i) IC children, defined as children with a diagnosis of primary or secondary immunodeficiency, including haematologic malignancies, neutrophil or lymphocyte defects (including HIV), asplenia, complement deficiencies, or those receiving immunosuppressive therapies (chemotherapy, long‐term corticosteroids, biologics, or post‐transplant immunosuppression); (ii) non‐immunocompromised (non‐IC) children with high‐risk conditions, defined as children with chronic underlying conditions not involving the immune system but known to increase the risk of severe COVID‐19 (e.g., congenital heart disease, chronic respiratory conditions, metabolic or neurological disorders); and (iii) a reference (RC) group of immunocompetent children without known high‐risk underlying conditions.

### 2.3 Variables of Interest and Outcomes

The primary outcomes of interest were: (i) SARS‐CoV‐2 infection, defined as the first laboratory‐confirmed positive SARS‐CoV‐2 RT‐PCR or antigen nasopharyngeal swab; (ii) COVID‐19‐related hospitalisation, defined as a hospital admission occurring within 10 days of a positive SARS‐CoV‐2 swab; and (iii) severe COVID‐19, defined as requiring admission to an intensive care unit (ICU), respiratory or circulatory support, or resulting in death. The 10‐day window was selected based on the typical timeline of COVID‐19 progression, as clinical deterioration and hospitalisation most commonly occur within 5–8 days from symptom onset [15]. This interval was considered sufficiently inclusive to capture hospitalisations temporally related to acute infection while limiting misclassification of unrelated admissions.

SARS‐CoV‐2 infection was identified through the first record of a positive SARS‐CoV‐2 test in each data source: In Norway, this was captured from the MSIS (based on RT‐PCR testing) and supplemented with confirmed diagnoses of COVID‐19 in primary and secondary care settings; in Veneto, Italy, infection was defined using the regional COVID‐19 testing registry, which includes both RT‐PCR and rapid antigen swab results.

COVID‐19‐related hospitalisations and severe outcomes were ascertained through manual review of discharge summaries by trained paediatricians (CD, GS, and EV) in Pedianet (Italy) and through ICD‐10 diagnostic/procedural codes or death records in Norway. (Table S3). Variables of interest included demographic characteristics (age, sex, socioeconomic status [area deprivation index in Italy and region of residence in Norway]), healthcare utilisation indicators (number of paediatric visits and number of antibiotic prescriptions in the year before 1 February 2020), clinical characteristics of SARS‐CoV‐2 infection (SARS‐CoV‐2 variant of concern, hospitalisation timing, ICU admission, ventilation, use of COVID‐19‐related therapies such as monoclonal antibodies or antivirals), and death, and COVID‐19 vaccination. The predominant circulating SARS‐CoV‐2 variant of concern at the time of children's infection onset was defined based on national surveillance data and the CovSPECTRUM platform [16], assigning each infection to a predominant circulating variant (ancestral, beta, and delta for the pre‐Omicron period, or Omicron for the Omicron period), as previously described [17]. The following timeframes were considered: (a) pre‐Omicron period (from 1 February 2020 to 15 November 2021) and (b) Omicron period (from 16 November 2021 to 28 February 2022).

### 2.4 Statistical Analysis

Descriptive statistics were used to summarise demographic and clinical characteristics across the three study groups (IC, non‐IC with high‐risk conditions, and RC). Categorical variables were reported as frequencies and proportions, and continuous variables as means with standard deviations (SD) or medians with interquartile ranges (IQR), as appropriate.

Cumulative incidence of SARS‐CoV‐2 infection was estimated using the Fine and Grey method, which accounts for competing risks (i.e., COVID‐19 vaccination), and stratified by exposure group. The proportional hazards assumption was assessed through Schoenfeld residuals.

To assess the association between immunocompromised status and SARS‐CoV‐2 infection, Fine‐Grey competing risk models were used to estimate adjusted sub‐hazard ratios (aHR) and 95% confidence intervals (CI). Comparisons were made between IC, non‐IC, and RC, including both IC vs. RC and IC vs. non‐IC. The models accounted for COVID‐19 vaccination as the competing event and adjusted for age, sex, area deprivation index (Italy) or region of residence (Norway), and healthcare utilisation in the year prior to follow‐up (number of paediatric visits and antibiotic prescriptions). COVID‐19‐related hospitalisation and severity among hospitalised children were assessed using adjusted log‐binomial regression models to estimate risk ratios (aRR) and corresponding 95% CI among those children who developed SARS‐CoV‐2 infection. Models were adjusted for the same covariates described above.

No formal statistical comparison was performed between countries, as data were analysed independently and with context‐specific model specifications.

SAS software, version 9.4 (SAS Institute Inc., Cary, NC, USA) and STATA version 18 (Stata‐Corp LP, College Station, TX, USA) were used for the analyses in Italy and Norway, respectively.

### 2.5 Ethics

The study was approved by the Pedianet Internal Scientific Committee and the Institutional Review Board of Società Servizi Pediatrici, Padova, Italy. In Norway, ethical approval was granted by the Regional Committees for Medical and Health Research Ethics (REK), and the use of registry data was authorised under the Norwegian Institute of Public Health's legal mandate for emergency preparedness. In both countries, all data were anonymised prior to analysis, and no identifiable information was accessible to the research team. Specifically, in Italy, data were anonymised and stored in a centralised database managed by Società Servizi Pediatrici, the legal owner of Pedianet. In Norway, the Data Protection Impact Assessment (DPIA no. 341884) was approved by the Norwegian Data Protection Services for Research and the University of Oslo. Given the retrospective nature of the study and the exclusive use of routinely collected health data, informed consent was not required.

## 3 Results

### 3.1 Study Populations Characteristics

A total of 29 520 children were included in the Italian cohort, of whom 201 (0.7%) were classified as IC, 5 144 (17.4%) as non‐IC with high‐risk conditions, and 24 175 (81.9%) as RC. In Norway, 851 517 children were included, with 4 862 (0.6%) classified as IC, 157 358 (18.5%) as non‐IC, and 689 297 (81.0%) as RC.

Table 1 summarises the sociodemographic characteristics of the Italian and Norwegian cohorts. In both cohorts, males were more prevalent in the IC and non‐IC groups, whereas in the RC group, the distribution of males and females was more balanced. In both cohorts, children classified as IC tended to be older than those in the RC group but younger than those classified as non‐IC children. Median age [IQR] was 7 years [4–9] in Italy and 8 years [4–11] in Norway for the IC group, compared to 6 years [4–9] in Italy and 8 years [5–11] in Norway for the RC group, and 8 years [5–10] in Italy and 8 years [4–11] in Norway for the non‐IC group. As expected, in both countries, IC children had higher healthcare utilisation, both in terms of primary care visits and antibiotic use.

**TABLE 1:** Baseline characteristics of the study population by country and clinical group of immunocompromised children (IC), non‐IC with high‐risk conditions, and reference children (RC).

	Italy			Norway		
	IC (*n* = 201)	non‐IC (*n* = 5 144)	RC (*n* = 24 175)	IC (*n* = 4 862)	non‐IC (*n* = 157 358)	RC (*n* = 689 297)
Median age in years, (IQR)	7 (4–9)	8 (5–10)	6 (4–9)	8 (4–11)	8 (5–11)	8 (4–11)
Age categories, *n* (%)						
1–2 years	24 (11.9)	316 (6.1)	3 433 (14.2)	538 (11.1)	14 470 (9.2)	77 682 (11.3)
3–4 years	31 (15.4)	606 (11.8)	4 294 (17.8)	717 (14.8)	21 279 (13.5)	103 828 (15.1)
5–11 years	127 (63.2)	3 589 (69.8)	14 725 (60.9)	2 532 (52.1)	87 946 (55.9)	373 882 (54.2)
≥ 12 years	19 (9.5)	633 (12.3)	1 723 (7.1)	1 075 (22.1)	33 663 (21.4)	133 905 (19.4)
Sex, *n* (%)						
Male	116 (57.7)	3 091 (60.1)	11 993 (49.6)	2 745 (56.5)	92 126 (58.6)	342 845 (49.7)
Female	85 (42.3)	2 053 (39.9)	12 182 (50.4)	2 117 (43.5)	65 232 (41.5)	346 452 (50.3)
COVID‐19 vaccination, *n* (%)						
No vaccination	119 (59.2)	2 888 (56.1)	15 460 (63.9)	2 682 (72.1)	114 329 (72.7)	536 412 (77.8)
At least 1 dose	82 (40.8)	2 256 (43.9)	8 715 (36.1)	1 180 (27.9)	43 029 (27.3)	152 885 (22.2)
Pre‐pandemic paediatric visits, *n* (%)						
0	14 (7.0)	463 (9.0)	3 002 (12.4)	197 (4.1)	11 485 (7.3)	221 915 (32.2)
1–5	85 (42.3)	2 877 (55.9)	14 056 (58.1)	1 496 (30.8)	75 531 (48.0)	365 694 (53.1)
6–10	51 (25.4)	1 231 (23.9)	4 913 (20.3)	1 378 (28.3)	42 067 (26.7)	77 530 (11.3)
≥ 11 visits	51 (25.4)	573 (11.1)	2 204 (9.1)	1 791 (36.8)	28 275 (18.0)	24 158 (3.5)
Pre‐pandemic antibiotic use, *n* (%)						
0	108 (53.7)	3 283 (63.8)	15 017 (62.1)	4 222 (86.8)	149 384 (94.9)	672 645 (97.6)
1–3 prescriptions	77 (38.3)	1 672 (32.5)	8 346 (34.5)	375 (7.7)	7 515 (4.8)	16 301 (2.4)
4–6 prescriptions	11 (5.5)	168 (3.3)	717 (3.0)	140 (2.9)	319 (0.2)	275 (< 0.1)
> 6 prescriptions	5 (2.5)	21 (0.4)	95 (0.4)	125 (2.6)	140 (0.1)	76 (< 0.1)

COVID‐19 vaccination coverage varied across groups and countries. In Italy, receipt of at least one dose was highest among non‐IC children (43.9%), followed by IC (40.8%) and RC (36.1%). In Norway, vaccination coverage was lower overall. At least one dose was received by 27.9% of IC, 27.2% of non‐IC, and 22.2% of RC children.

### 3.2 SARS‐CoV‐2 Infection

**FIGURE 1:**
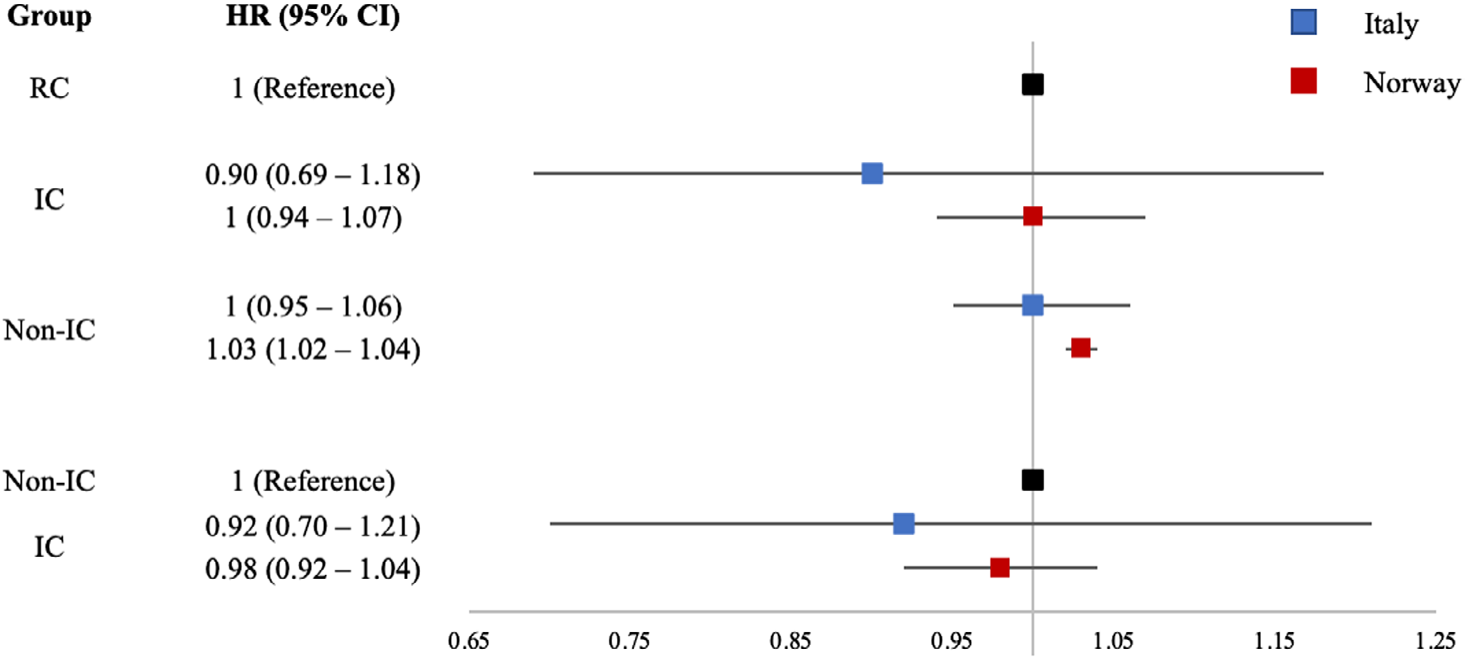
Risk of SARS‐CoV‐2 infection among children with immunocompromising conditions (IC) compared with non‐immunocompromised children with other risk factors for severe COVID‐19 (non‐IC) and with healthy children (i.e., reference children, RC) in Italy and Norway.

In Italy, 10 551 of 29 520 children (35.7%) had a laboratory‐confirmed SARS‐CoV‐2 infection (Table 2). The proportion of cases was similar across groups: 71 of 201 (35.3%) IC children, 1 849 of 5 144 (35.9%) non‐IC, and 8 631 of 24 175 (35.7%) RC children. In Norway, 252 226 of 851 517 children (29.6%) had a laboratory‐confirmed SARS‐CoV‐2 infection. As in Italy, the proportions were comparable across groups: 1 471 of 4 862 (30.3%) IC, 48 832 of 157 358 (31.0%) non‐IC, and 201 923 of 689 297 (29.3%) RC children (Table 2). The distribution of SARS‐CoV‐2 infections across variant of concern circulation periods (pre‐Omicron vs. Omicron) was similar among all groups in both countries, with most infections occurring during the Omicron period (Table 2). In Italy, IC children had a lower risk of SARS‐CoV‐2 infection compared to RC (aHR = 0.90, 95% CI: 0.69–1.18) and non‐IC children (aHR = 0.92, 95% CI: 0.70–1.21). Likewise, non‐IC children had a risk comparable to that of the RC group (aHR = 1.00, 95% CI: 0.95–1.06) (Figure 1). Similarly, in Norway, IC children had no increased risk of infection compared to RC (aHR = 1.00, 95% CI: 0.94–1.07) or non‐IC children (aHR = 0.98, 95% CI: 0.92–1.04). Non‐IC children had a slightly higher risk of SARS‐CoV‐2 infection than RC (aHR = 1.03, 95% CI: 1.02–1.04) (Figure 1).

**TABLE 2:** Distribution of SARS‐CoV‐2 infections by country and clinical group: Immunocompromised children (IC), non‐IC with high‐risk conditions, and reference children (RC).

	Italy			Norway		
	IC (*n* = 201)	non‐IC (*n* = 5 144)	RC (*n* = 24 175)	IC (*n* = 4 862)	non‐IC (*n* = 157 358)	RC (*n* = 689 297)
SARS‐CoV‐2 infection, *n* (%)	71 (35.3)	1 849 (35.9)	8 631 (35.7)	1 471 (30.3)	48 832 (31.0)	201 923 (29.3)
SARS‐CoV‐2 variant of concern circulation period, *n* (%)						
Pre‐Omicron	24 (33.8)	782 (42.3)	3 581 (41.5)	424 (28.2)	12 468 (25.5)	54 055 (26.8)
Omicron	47 (66.2)	1 067 (49.6)	5 050 (58.5)	1 047 (71.2)	36 364 (74.5)	147 868 (73.2)
COVID‐19‐related hospitalisation, *n* (%)	< 5 (< 1.5)	19 (1.0)	78 (0.9)	8 (0.5)	82 (0.2)	98 (0.1)

### 3.3 COVID‐19‐Related Hospitalisation and COVID‐19 Severity

In Italy, among 10 551 children with laboratory‐confirmed SARS‐CoV‐2 infection, 98 (0.93%) were hospitalised for COVID‐19. Hospitalisation occurred in < 1.5% (< 5 out of 71) of IC children, 1.0% (19 out of 1 849) of non‐IC children, and 0.9% (78 out of 8 631) of RC children. In the Norwegian cohort, 0.5% (8 out of 1 471) of IC children were hospitalised, compared with 0.2% (82 out of 48 832) in the non‐IC group and 0.1% (98 out of 201 923) in RC children (Table 2).

**FIGURE 2:**
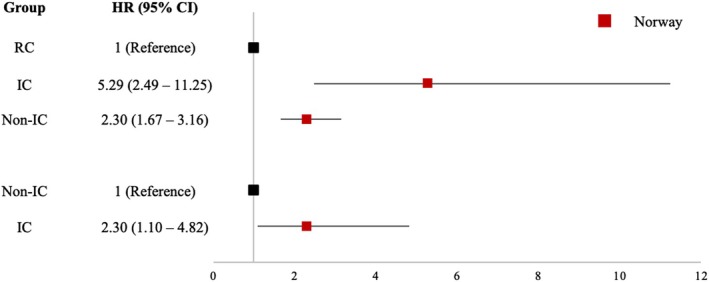
Risk of COVID‐19‐related hospitalisation among children with immunocompromising conditions (IC) compared with non‐immunocompromised children with other risk factors for severe COVID‐19 (non‐IC) and with healthy children (i.e., reference children, RC) in Norway.

In Italy, due to the very small number of hospitalised IC children (*n* < 5), no statistical modelling was conducted. In Norway, an elevated risk of COVID‐related hospitalisation among children with underlying conditions, including immunocompromising and non‐immunocompromising conditions, was confirmed. Compared to RC children, the aRR was 5.29 (95% CI: 2.49–11.25) for IC children and 2.30 (95% CI: 1.67–3.16) for non‐IC children. When directly comparing IC to non‐IC children, the aRR was 2.30 (95% CI: 1.10–4.82), indicating a significantly higher risk of hospitalisation among IC children (Figure 2).

Severe COVID‐19 was rare in both cohorts. No severe cases were reported in Italy. In Norway, five cases were documented, including both IC and non‐IC children.

## 4 Discussion

In this large, population‐based study across two paediatric cohorts in Italy and Norway, we found that IC children had a similar risk of SARS‐CoV‐2 infection compared with both children with and without non‐immunocompromising high‐risk conditions.

However, IC children showed a higher risk of COVID‐19‐related hospitalisation compared with non‐IC and RC children in both cohorts, despite the very low number of severe cases, none in Italy and < 5 in Norway. These findings are consistent with previous paediatric studies reporting that IC status has been associated with higher hospitalisation rates but not with increased risk of severe disease [18]. Prior research in hospitalised populations has shown similar patterns, often demonstrating lower severity in IC children compared with their immunocompetent peers [6, 7, 19]. In the US COVID‐NET study, which assessed predictors of severe outcomes among children hospitalised with COVID‐19 as the primary reason for admission, immunocompromising conditions were associated with a significantly lower adjusted risk of severe disease compared with immunocompetent peers (aRR = 0.68; 95% CI 0.60–0.78) [6]. Similarly, in the large individual‐patient meta‐analysis by Harwood et al., the odds of critical care admission or death increased with the number of comorbidities, but malignancy and other immunocompromising conditions were not linked to higher severity (OR = 0.85; 95% CI 0.17–4.21) [19]. In our previous Canadian multicentre surveillance study, IC children hospitalised for COVID‐19 had a lower risk of severe disease than non‐IC peers (aRR = 0.46; 95% CI 0.32–0.65) [7].

Our results confirm and extend these observations at a population level, adding to existing population‐based evidence that IC children may experience higher hospitalisation rates despite not developing more severe disease compared to immunocompetent children [2]. Similarly, a meta‐analysis reported lower rates of severe COVID‐19 in immunodeficient and immunosuppressed paediatric and adult populations compared to the general population [20]. A similar pattern has been documented for other viral infections, including influenza, in which IC children are disproportionately represented among hospitalised cases despite having similar or lower severity [21]. For instance, data from influenza surveillance in Canada, the US, and Australia have consistently shown lower risks of intensive care and respiratory support need in hospitalised IC children compared to immunocompetent children [22, 23, 24].

Nevertheless, the literature is not entirely consistent. A systematic review and meta‐analysis found a trend toward more severe outcomes among immunocompromised children and young people [16], echoing findings in adult populations [3, 25]. However, many of these studies were conducted in hospital‐based settings, including patients with more severe conditions, and thus do not necessarily reflect the risk in outpatient settings. Indeed, another meta‐analysis found that rates of ICU admission and death among children with COVID‐19 were markedly lower in community‐based studies compared to hospital‐based studies [26].

The IC paediatric population is also highly heterogeneous, comprising a wide spectrum of immune deficiencies and immunosuppressive treatments that may differentially influence incidence outcome discrepancies across studies. Some studies focusing on narrowly defined high‐risk subgroups such as children receiving chemotherapy—have shown greater severity [26], highlighting the need for disaggregated analysis of IC subpopulations.

Our study addresses a critical gap by leveraging population‐level, real‐world data from two national paediatric cohorts with high coverage, allowing for robust comparisons across healthcare systems. However, due to the very low number of severe cases, we were unable to evaluate the risk of severe COVID‐19 among hospitalised IC, non‐IC, and RC children with SARS‐CoV‐2 infection.

Furthermore, while we harmonised definitions across cohorts, several limitations should be noted. First, immunocompromising and non‐immunocompromising conditions were defined using diagnostic codes and free‐text from electronic health records and nationwide health registries, which may result in under‐ascertainment or misclassification of certain conditions. Second, due to the low number of severe outcomes, we were underpowered to explore heterogeneity within IC subgroups (e.g., transplant recipients or children receiving chemotherapy). Third, real‐world data do not capture information on non‐pharmacological preventive measures, which may have played an important role in modulating infection risk across groups. Lastly, while Pedianet is broadly representative, it was limited to the Veneto region, potentially affecting generalizability across the entire country. Additionally, especially during the early stages of the pandemic, misclassification of exposure status may have occurred, potentially leading to an underestimation of COVID‐19‐related hospitalisations. Moreover, COVID‐19 hospitalisations were defined as admissions occurring within 10 days of a positive SARS‐CoV‐2 test. Although this approach was chosen to reflect the typical timeline of clinical worsening and minimise under‐ascertainment of COVID‐19‐related admissions, some hospitalisations within this window may have represented incidental SARS‐CoV‐2 detections, potentially introducing non‐differential misclassification.

In summary, in two large population‐based paediatric cohorts, IC children had similar infection risk but higher hospitalisation rates than immunocompetent children with and without underlying risk factors, while severe cases were rare in both countries. Further studies are needed to clarify whether this pattern reflects differences in disease severity, healthcare‐seeking behaviour, or clinical management practices.

## Author Contributions

**Costanza Di Chiara:** conceptualization, investigation, data curation, writing – original draft, project administration. **Nhung T. H. Trinh:** data curation, formal analysis, resources, methodology, writing – review and editing, investigation. **Riccardo Boracchini:** methodology, formal analysis, writing – review and editing, data curation. **Arianna Giugni:** methodology, formal analysis, writing – review and editing, data curation. **Giulia Sturniolo:** data curation and data extraction, writing – review and editing. **Ali Judd:** writing – review and editing, visualization, supervision. **Marthe Le Prevost:** writing – review and editing, visualization. **Claire Thorne:** writing – review and editing, visualization. **Pia Hardelid:** writing – review and editing, visualization. **Carlo Giaquinto:** writing – reviewing and editing, visualization, supervision, resources. **Daniele Donà:** writing – reviewing and editing, supervision. **Angela Lupattelli:** data curation, formal analysis, resources, supervision. **Anna Cantarutti:** conceptualization, investigation, formal analysis, resources, methodology, writing – reviewing and editing, supervision. All authors have reviewed and approved the final manuscript.

## Funding

This work is partially supported by the VERDI project (101045989), which is funded by the European Union. Views and opinions expressed are, however, those of the author(s) only and do not necessarily reflect those of the European Union or the European Health and Digital Executive Agency. Neither the European Union nor the granting authority can be held responsible for them. This work was supported by the European Union, VERDI project (101045989).

## Ethics Statement

Approval of the study and access to the database were granted by the Internal Scientific Committee of So.Se.Te. Srl, the legal owner of Pedianet, Padova (Italy). In Norway, the Regional Committee for Medical and Health Ethics of South/East Norway (no. 285687 on 15 September 2021) approved the study. The Norwegian Data Protection Services for research and the University of Oslo approved the Data Protection Impact Assessment—DPIA (no. 341884). Written informed consent to participate in this study was not required from the participants or the participants' legal guardians/next of kin in accordance with Norwegian national legislation and the institutional requirements.

## Conflicts of Interest

The authors declare no conflicts of interest.

## Supporting information


**Table S1:** Immunocompromising condition categories and ICD‐9‐CM, ICD‐10‐CM, and ICPC‐2 codes [1–4].
**Table S2:** Non‐immunocompromising condition categories and ICD‐9‐CM, ICD‐10‐CM, and ICPC‐2 codes.
**Table S3:** SARS‐CoV‐2 infection and respiratory tract infection (including COVID‐19) related hospitalisation ICD‐10‐CM codes.

## Data Availability

The data that support the findings of this study are available from the corresponding author upon reasonable request.
