# Supplementary material for: Risk of SARS‐CoV‐2 Infection and Hospitalisation in Immunocompromised Children: A Population‐Based Cohort Study in Italy and Norway
Source: Acta Paediatr. 2026 Mar 23;115(7):1451–60. doi: 10.1111/apa.70509 (PMC13250967; doi:10.1111/apa.70509)
Supplement: Supplementary file 1 — Table S1: Immunocompromising condition categories and ICD‐9‐CM, ICD‐10‐CM, and ICPC‐2 codes [1–4]. Table S2: Non‐immunocompromising condition categories and ICD‐9‐CM, ICD‐10‐CM, and ICPC‐2 codes. Table S3: SARS‐CoV‐2 infection and respiratory tract infection (including COVID‐19) related hospitalisation ICD‐10‐CM codes. [file APA-115-1451-s001.docx]

**Supplementary materials**

*Table S1*. Immunocompromising condition categories and ICD-9-CM, ICD-10-CM, and ICPC-2 codes [1-4].

| *Immunocompromising condition categories* | Conditions | | ICD-9-CM code (Italy) | ICD-10 code (Norway) | ICPC-2 code (Norway) |
| --- | --- | --- | --- | --- | --- |
| *Humoral, cellular, and combined immunity deficiencies* | Humoral immunodeficiency (including Common variable immunodeficiency) |  | 279.0* | D80, D83 |  |
|  | Cellular immunodeficiency |  | 279.1* | G11.3 |  |
|  | Combined humoral and cellular immunity deficiencies (including Wiskott-aldrich syndrome) |  | 279.2*, 334.8* | D81, D82 |  |
| *Other specified and unspecified disorders involving the immune mechanism* |  |  | 279.3*, 279.8*, 279.9* | D84 |  |
| *HIV infection* | HIV infection |  | 042, V08, 079.5* | B20 | B90 |
| *Neoplasms* | Malignant neoplasms of lymphatic and hematopoietic tissue |  | 200.**-208.**, 230.**-239.** | C81-C96, D45, D46, D47 | B72, B73, B74 |
|  | Malignant neoplasm | Malignant neoplasm of lip, oral cavity, and pharynx | 140.**-149.**, | C00-C14 | D77 |
|  |  | Malignant neoplasm of digestive organs and peritoneum | 150.**-159.** | C15-C26 | D74, D75, D76, D77 |
|  |  | Malignant neoplasm of respiratory and intrathoracic organs | 160.**-165.** | C30-C39 | R85, H75, R84, B74, K72, A79 |
|  |  | Malignant neoplasm of bone, connective tissue, skin, and breast | 170.**-176.**, | C40-C50 | L71, S77, R85, D77, K72, A79, S77, B74, N74, X76, Y78 |
|  |  | Malignant neoplasm of genitourinary organs | 179.**-189.** | C51-C68 | X77, X75, W72, Y78, Y77, U75, U77, U76 |
|  |  | Malignant neoplasm of other and unspecified sites | 190.**-199.**, | C69-C76, C80 | F74, N74, T71, T73, A79 |
|  | Other disorders of blood and blood-forming organs, including spleen disorders |  | 282.**, 284.**, 288.**, 289.**, 759.0 | D55-D59, D70, D71, D72, D73, D75-D77, Q89.01 | B82, B78, B84, T80 |
| *Transplant* | Bone marrow or hematopoieric stem cell transplant |  | 41.0* | Z94.8 |  |
|  | Solid Organ Transplant |  | V42.* | Z94.0, Z94.1, Z94.2, Z94.3, Z94.4, Z94.9 |  |
| *Chemotherapy* |  |  | V58.11, V58.12 | Z51.1, Z51.11, Z51.12 |  |

[1] Angel A. Justiz Vaillant; Ahmad Qurie. Stat Pearls Immunodeficiency. Last update: July 8, 2022. Last access: July, 11, 2023. Bookshelf ID: NBK500027. PMID: 29763203. Available at: <https://www.ncbi.nlm.nih.gov/books/NBK500027/#:~:text=Primary%20immunodeficiencies%20leading%20to%20T,and%20interleukin%2D12%20receptor%20deficiency>.

[2] <https://www.cdc.gov/coronavirus/2019-ncov/hcp/clinical-care/underlyingconditions.html>

[3] https://www.canada.ca/en/public-health/services/diseases/2019-novel-coronavirus-[infection/guidance-documents/signs-symptoms-severity.html#a3](https://www.canada.ca/en/public-health/services/diseases/2019-novel-coronavirus-infection/guidance-documents/signs-symptoms-severity.html#a3) Accessed on April 24, 2023

[4] Norwegian Institute of Public Health. Coronavirus vaccine – information for the public. [Coronavirus vaccine - NIPH (fhi.no)](https://www.fhi.no/en/id/corona/coronavirus-immunisation-programme/coronavirus-vaccine/#about-risk-groups-and-childrenadolescents-with-underlying-conditions) Accessed on February 14, 2024

*Table S2*. Non-immunocompromising condition categories and ICD-9-CM, ICD-10-CM, and ICPC-2 codes.

Non-immunocompromising conditions were classified into different underlying condition categories (i.e., cardiovascular, respiratory, neurocognitive, metabolic, and other conditions) based on CDC criteria [1]. Within each underlying condition category, only those conditions that were recognized as risk factors for severe COVID-19 were included and considered for the analysis [1-3].

| *Non-immunocompromising condition categories* | Conditions | | ICD-9-CM code (Italy) | ICD-10-CM code (Norway) | ICPC-2 code (Norway) |
| --- | --- | --- | --- | --- | --- |
| *Cardiovascular conditions* | Chronic cardiac diseases | Chronic rheumatic heart disease | 393.**-398.** | I05-I09 | K71 |
|  |  | Cardiomyopathy | 425.** | I42, I43 | K73, K84 |
|  |  | Cardiomegaly | 429.3 | I51.7 | K84 |
|  |  | Cardiac arrest | 427.5 | I46 | K84 |
|  |  | Heart failure | 428.** | I50 | K77 |
|  |  | Myocardial infarction | 410-414, 429.71-79, 429.81-89, 429.9*, | I21, I25 | K75, K76 |
|  | Congenital | Congenital heart diseases | 746.**, 747.** | Q20-Q28 | K73 |
|  | Aerhytmias | Cardiac dysrhythmias | 427.** | I44, I45, I47, I48, I49 | K84, K78, K79 |
| *Cerebrovascular conditions* | Hemorrhagic conditions | Intracerebral hemorrhage | 430.**, 431.**, 432.0*-1*, 432.9*, | I60, I61, I62, I63 | K90 |
|  | Ischemic conditions | Occlusion and stenosis of precerebral and cerebral arteries, Transient cerebral ischemia | 433.**, 434.**, 435.**, 436.**, 437.** | I65, I66, I67, G45 | K91, K89 |
| *Respiratory conditions* | Chronic obstructive pulmonary disease and allied conditions | limited to: bronchiectasis, chronic bronchitis, BPCO, interstitial lung disease, and asthma | 490.**-496.**, 516.30-37, 516.61-69, 518.1*, | J40-J47, J80-J84, J98 | R78, R79, R95, R96, R99 |
|  | Cystic fibrosis |  | 277.0* | E84 |  |
|  | Diseases of pulmonary circulation |  | 415.**. 416.**, 417.** | I26-I28 | K93, K82 |
|  | Pulmonary tuberculosis |  | 010.**-012.** | A14 |  |
| *Neurocognitive conditions* | Disabilities, personality disorders, and behavioural problems | Down syndrome, psychosis, hyperkinetic syndrome of childhood, development delays, intellectual disabilities, mental and behavioural problems, | 758.0*, 290.**-299.**, 300.**, 301.**, 311.**, 313.**, 314.**, 315.**, 317.**-319.**, V40.**, | Q90, F20-F29, F30-F39, F40-F48, F50-F59, F70-F79, F80-F89, F90-F98, F99 | A90, P72, P98, P73, P76, P79, P74, P76, P02, P82, P75, P29, P75, P78, P99, P86, P29, P06, P07, P08, P76, P98, P18, P85, P24, P81, P22, P10 |
|  | Neurological conditions | Hereditary and degenerative diseases of the central nervous system, spinal cord injuries, epilepsy and cerebral palsy, inflammatory and demyelinating neuropathies, myoneural disorders, muscular dystrophies, myasthenia gravis, and other myopathies, spina bifida | 330.**, 333.**, 334.**, 335.**, 336.**, 337.**, 343.**, 345.**, 357.0*, 358.**-359.**, 952.**, 767.4*, 741.**, | G24, G35-G37, G40 , G60, G70-G73, G80, G94, G96, S14.**, S24.**, S34.**, G95, Q05 | N99, N86, N99, N88, N94, N81, N85 |
| *Metabolic conditions* | Diabetes and associated conditions | Diabetes mellitus, type 1 and type 2, chetoacidosis, coma | 250.**, 251.** | E08.00-E08.9, E10, E11, E13 | T89, T90 |
|  | Obesity and metabolic syndrome | Overweight and obesity, and metabolic syndrome | 278.00-.03, 277.7 | E66 | T82 |
| *Other conditions* | Renal conditions | Chronic kidney disease (CKD) and renal failure | 585.**, 586.** | N18, N19 | U99 |
|  | Liver conditions | Chronic liver diseases and cirrhosis, autoimmune hepatitis | 570.**, 571.40-.49, 571.5*, 571.6*-.9*, 572.**, 573.** | K72, K73, K74, K75, K76, K77 | D97 |

[1] <https://www.cdc.gov/coronavirus/2019-ncov/hcp/clinical-care/underlyingconditions.html>

[2] <https://www.canada.ca/en/public-health/services/diseases/2019-novel-coronavirus-infection/guidance-documents/signs-symptoms-severity.html#a3> accessed on November 10, 2023.

[3] Norwegian Institute of Public Health. Coronavirus vaccine – information for the public. [Coronavirus vaccine - NIPH (fhi.no)](https://www.fhi.no/en/id/corona/coronavirus-immunisation-programme/coronavirus-vaccine/#about-risk-groups-and-childrenadolescents-with-underlying-conditions) Accessed on February 14, 2024

*Table S3*. SARS-CoV-2 infection and respiratory tract infection (including COVID-19) related hospitalization ICD-10-CM codes.

| *Study Outcome* | ICD-10-CM code (Norway) | ICPC-2 code (Norway) |
| --- | --- | --- |
| *SARS-CoV-2 infection* | U071 | R992 |
| *Respiratory tract infection (including COVID-19) related hospitalization* | J12.8: pneumonia  J18: pneumonia  J20.8: acute bronchitis  J22: acute lower respiratory infection  J45.0: Asthma  J45.8: allergic asthma  J45.9: unspecified asthma  J96.0: respiratory failure  J98.8: Other specified respiratory disorders | NA |
| *Intensive care unit (ICU) admission* | NA | NA |
| *Ventilation* | GXAV01 using NCMP code system for procedure | NA |
| *Death within 10 days after SARS-COV-2 diagnosis* | based on available death date linked records | NA |
